# Bedside lung ultrasonography by emergency department residents as an aid for identifying heart failure in patients with acute dyspnea after a 2-h training course

**DOI:** 10.1186/s13089-021-00207-9

**Published:** 2021-02-09

**Authors:** Mohamed Amine Msolli, Adel Sekma, Maryem Ben Marzouk, Wael Chaabane, Khaoula Bel Haj Ali, Lotfi Boukadida, Nasri Bzeouich, Imen Gannoun, Imen Trabelssi, Kamel Laaouiti, Mohamed Habib Grissa, Kaouthar Beltaief, Zohra Dridi, Asma Belguith, Mehdi Methamem, Wahid Bouida, Riadh Boukef, Hamdi Boubaker, Semir Nouira, the GREAT Network

**Affiliations:** 1 Emergency Department, Fattouma Bourguiba University Hospital, 5000 Monastir, Tunisia; 2 Emergency Department, Sahloul University Hospital, 4011 Sousse, Tunisia; 3 Research Laboratory LR12SP18, University of Monastir, 5019 Monastir, Tunisia; 4 Cardiology Department, Fattouma Bourguiba University Hospital, 5000 Monastir, Tunisia; 5 Department of Preventive Medicine, Fattouma Bourguiba University Hospital, 5000 Monastir, Tunisia; 6 Emergency Department, Farhat Hached University Hospital, 4031 Sousse, Tunisia

**Keywords:** Lung ultrasonography, B-lines, Congestive heart failure, Diagnosis, Accuracy, Reproducibility

## Abstract

**Background:**

Ultrasonographic B-lines have recently emerged as a bedside imaging tool for the differential diagnosis of acute dyspnea in the Emergency Department (ED). However, despite its simplicity, LUS has not fully penetrated emergency department. This study aimed to assess the accuracy and reproducibility of ultrasonographic B-lines performed by emergency medicine (EM) residents for the diagnosis of congestive heart failure (CHF) in patients admitted to ED for acute dyspnea.

**Patients and methods:**

This is a cross-sectional prospective study conducted between January 2016 and October 2017 including patients aged over 18 years admitted to ED for acute dyspnea. At admission, two consecutive bedside LUS study were performed by a pair of EM residents who received a 2-h course for recognition of sonographic B-lines to determine independently B-lines score and B-profile pattern. All participating sonographers were blinded to patients’ clinical data. B-lines score ≥ 15 or a B-profile pattern was considered as suggestive of CHF. The final leading diagnosis was assessed by two expert sonographers, who were blinded to the residents’ interpretations, based on clinical findings, chest X-ray, brain natriuretic peptide, cardiac and lung ultrasound testing. Accuracy and agreement of B-lines score and B-profile pattern were calculated.

**Results:**

We included 700 patients with a mean age of 68 ± 12.6 years and a sex ratio (M/F) of 1.43. The diagnosis of CHF was recorded in 371 patients (53%). The diagnostic performance of B-lines score at a cut-off 15 and B-profile pattern was, respectively, 88% and 82.5% for sensitivity, 75% and 84% for specificity, 80% and 85% for positive predictive value, 84% and 81% for negative predictive value. The area under receiver operating characteristic curve was 0.86 [0.83–0.89] and 0.83 [0.80–0.86], respectively, for B-lines score and B-profile pattern. There was an excellent agreement between residents for the diagnosis of CHF using both scores (kappa = 0.81 and 0.85, respectively, for ordinal scale B-lines score and B-profile pattern).

**Conclusion:**

Lung ultrasound B-lines assessment has a good accuracy and an excellent reproducibility in the diagnosis of CHF in the hand of EM residents following a short training program.

*Trial registration* Name of the registry: clinicaltrials.gov; Trial registration number: NCT03717779; Date of registration: October 24, 2018 ‘Retrospectively registered’; URL of trial registry record: clinicaltrials.gov

## Introduction

Acute dyspnea is a common clinical emergency and a leading cause of hospital admissions [1]. While the differential diagnosis is broad, congestive heart failure (CHF) is one of the most frequent causes that can be difficult to differentiate from other etiologies. Although immediate and accurate diagnosis is critical, available diagnostic modalities of CHF among dyspneic patients, lack either specificity or sensitivity [2–4]. Echocardiography was shown to be pivotal in the diagnostic workup of CHF, but such facility requires high skills and is not always available in many emergency departments [5, 6]. Recently, lung ultrasound (LUS) has emerged as a promising alternative tool that can be performed by novice sonographers [7–13]. This easy non-invasive bedside method provides rapid diagnostic information allowing an earlier and targeted treatment. Consequently, LUS is increasingly used in clinical practice particularly in acute care settings [10]. Nonetheless, before accepting the widespread use of LUS, there is still need to assess its accuracy and reproducibility in the hand of non-experts.

The purpose of our study is to evaluate the accuracy and reproducibility of B-lines testing assessed by emergency medicine (EM) residents after 2-h training in the diagnosis of CHF in patients admitted to the emergency department with acute dyspnea.

## Patients and methods

### Patients

This is prospective cross-sectional study conducted in the Emergency Department (ED) of three University Hospitals (Fattouma Bourguiba University Hospital, Sahloul University Hospital, and Farhat Hached University Hospital, Tunisia) from January 2016 to October 2017.

A convenience sampling approach, including all patients admitted to the ED for acute dyspnea as chief complaint, was used. Exclusion criteria were: age less than 18 years, impossibility to give consent to participate in the study, post-traumatic dyspnea, pregnant women, and need for endotracheal intubation or inotropic drugs patients who were deemed too unstable for sonography by the treating team were also excluded.

### Methodology

All eligible patients underwent a complete physical examination. Blood pressure, heart rate, and pulse oximetry were measured and oxygen was delivered by face mask as needed. Research associates collected the following data: name, age, sex, previous medical history, ongoing treatment, and physical examination findings. The following additional tests were performed for all included patients: blood gas, hemoglobin, serum creatinine, BNP, electrocardiogram, chest X-ray, and echocardiogram. Lung ultrasonography was performed by EM residents using two ultrasound machines (Philips EnVisor C, Nederland; SonoSite M-Turbo, Sonosite Inc., Bothell, WA) and broadband curved array probes (3.5–5 MHz). The study period overlapped one and half academic year in three university hospitals, so a total of 40 residents were eligible to participate. ED residents were appointed to carry out this examination less than 4 h following patients’ admission. None of the ED residents used LUS for the assessment of B-lines prior to the study. All participating residents were previously attended a 2-h training session with at least 10 clinical tests supervised by a certified emergency physicians who had accomplished a full mentoring program for “Ultra-Sound Life Support”. The first 30 min of the training course included basic ultrasound physics, use of ultrasound equipment, probe positioning, and lung ultrasound interpretation (A-lines, B-lines, consolidation, lung sliding, lung pulse, and miscellaneous artifacts). In the second 30 min, real-time LUS was performed in healthy volunteers describing the technique and findings. The rest of the training was hands-on training on actual patients. Trainees had to identify the presence of lung sliding, A-lines, B-lines and consolidation.

**Fig. 1:**
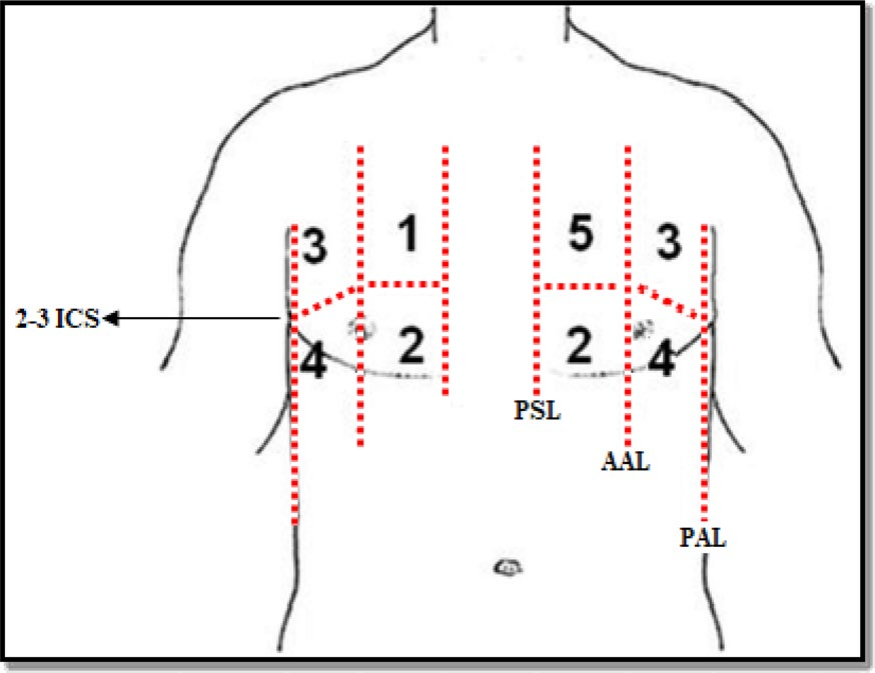
The four chest ultrasound areas per side. Areas 1 and 2 denote the upper anterior and lower anterior chest areas, respectively. Areas 3 and 4 denote the upper lateral and basal lateral chest areas, respectively. *PSL* parasternal line, *AAL* anterior axillary line, *PAL* posterior axillary line, *ICS* intercostal space

**Fig. 2:**
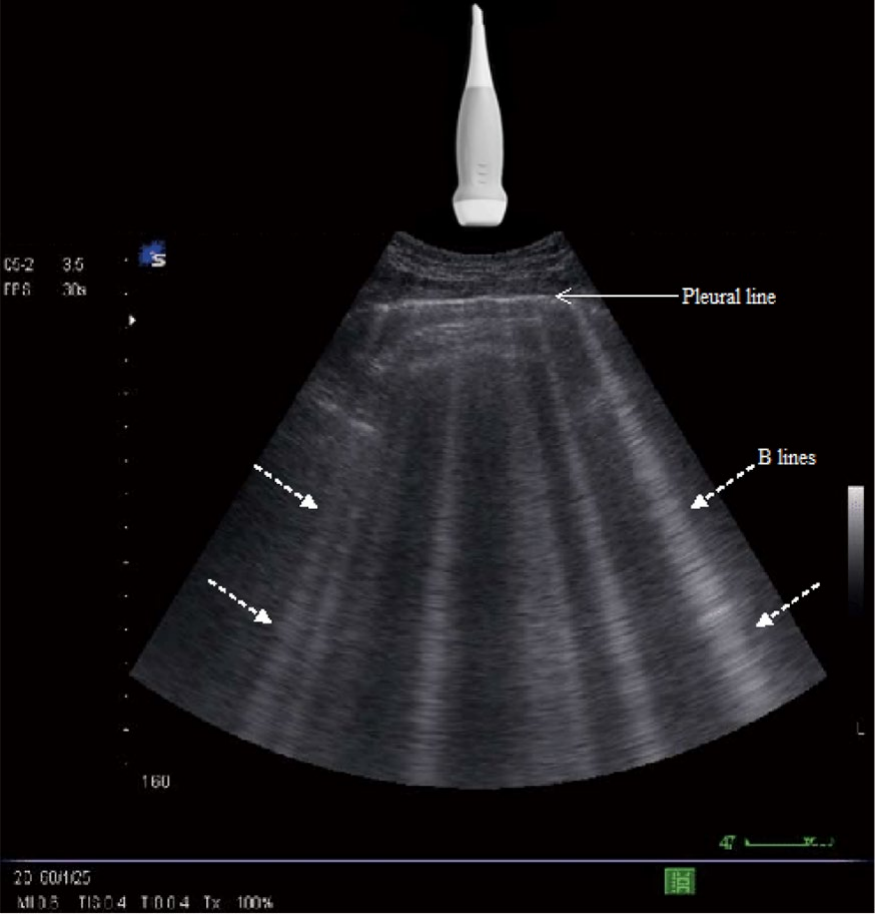
B-lines represented by vertical hyperechoic images starting from the pleural line and extending to the whole ultrasound field (discontinuous arrows)

For each patient, two LUS tests were performed by two independent residents who were not aware of patient's clinical data and did not participate in the patient’s management. We recorded the ED residents’ interpretation and images were recorded for each LUS study for later expert review. To not break the blind protocol, patients were asked to not provide information on their medical history to the operators during LUS. Patients were placed in a semi-recumbent or supine position depending on their respiratory tolerance. For each side of the chest, 4 zones have to be assessed (Fig. 1): 2 anterior and 2 lateral. The anterior chest wall was delineated from the sternum to the anterior axillary line and was subdivided into upper and lower halves (approximately from clavicle to the second–third intercostal spaces and from the third space to diaphragm). The lateral chest was delineated from the anterior to the posterior axillary line and was subdivided into upper and basal halves. The operator was asked to calculate the B-lines score which is the sum of the B-lines found in both sides (8 zones) [14]; the intercostal space with the greatest number of B-lines within each zone was used for scoring. B-line was defined as a vertical bright echogenic bundle with a narrow basis, spreading from the transducer to the deepest part of the screen (Fig. 2). For B-lines that were wide or confluent, the score was determined by assessing the percentage of the rib space occupied by B-lines and dividing it by ten [10].

According to the study of Gargani et al. the B-lines score is suggestive of CHF when it is ≥ 15 [15]. The probability of CHF was also expressed according to the following ordinal scale: unlikely if B-lines score < 15, likely if B-lines score is between 16 and 29, and very likely if B-lines score ≥ 30. The operator also had to assess the presence or absence of B-profile pattern which is suggestive of CHF according to Lichtenstein criteria [8]. B-profile pattern was defined as such if two or more lung zones per side were positive. A lung zone was positive if three or more B-lines were identified. The final leading diagnosis of dyspnea was assessed by two independent senior EM physicians after reviewing the entire medical record of each patient it was based on: (1) the clinical presentation (severe shortness of breath, worsening dyspnea, orthopnea, paroxysmal nocturnal dyspnea, coughing up or wheezing with white or pink blood-tinged phlegm, foamy mucus), and the physical exam findings (pulmonary congestion and/or peripheral edema, rales, crackles); (2) the diagnostic tests’ results including chest X-ray (pulmonary venous congestion, pleural effusion, interstitial or alveolar edema and cardiomegaly), echocardiography (structural or functional cardiac abnormalities), brain natriuretic peptide (BNP > 300 pg/mL, or NT-proBNP > 1200 pg/mL), the saved images of LUS study, treatment, and outcome [4]. In case of a disagreement, a third senior physician was consulted and adjudicated the case. All senior physicians participating in the study were masked to LUS results. Informed consent was obtained in all the patients before the start of the protocol.

### Statistical analysis

Prior to enrollment, a power analysis was performed to determine the sample size needed. Assuming an alpha of 0.05 and a desired precision of 0.07, we calculated a sample size of 502 patients required if we considered that the estimated prevalence of CHF is 25% and the targeted sensitivity and specificity would both be 0.80.

After analysis of normality distribution, variables were expressed by the arithmetic mean and standard deviation (SD) or the median and the 95% confidence interval (or interquartile range). Comparison between patients with CHF (HF group) and those without CHF (non-HF group) was performed by Student’s t-test for continuous variables and Chi-2 test for categorical variables. The difference was considered statistically significant for values of *p* ≤ 0.05. Discrimination power of the assessed models was studied by the area under the receiver operating characteristic (ROC) curve. An area under curve (AUC) = 1 represents a perfect test; an area of 0.5 represents a worthless test (random prediction), and an area greater than 0.70 means that accuracy of the diagnostic test is at least fair. For the assessment of diagnostic accuracy of B-lines, the scanning order was randomly determined according to an electronic randomization. Agreement between residents’ interpretation was assessed by kappa agreement index for qualitative indices (B-lines score as ordinal scale, and B-profile pattern recorded dichotomously as present or absent). Agreement was considered “low” when kappa value was less 0.40, “fair” from 0.41 to 0.60, “good” from 0.61 to 0.80 and “excellent” from 0.81 to 1. For the B-lines score, the Bland and Altman plot was constructed. A good match was defined when the differences between B-lines score pairs is around the average line and between the lines of − 2 and + 2 SD. The data obtained in this study were collected, recorded and analyzed using SPSS computer software version 18.0 (Chicago, IL).

## Results

**Fig. 3:**
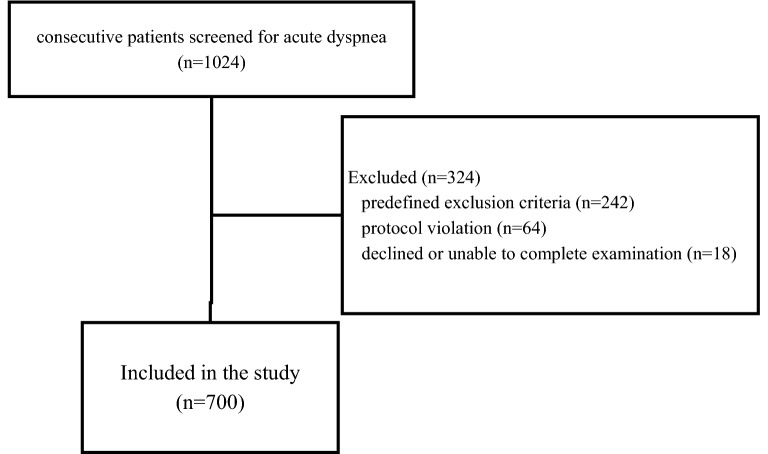
Summary of patients’ selection

During the study period, 1024 patients with acute dyspnea were screened. Two hundred forty-two patients were excluded for one or more predefined exclusion criteria; additional 64 patients were excluded for blind protocol violation, and 18 declined or were unable to tolerate a complete examination (Fig. 3). The characteristics of the remaining 700 patients are outlined in Table 1. Four hundred twelve patients (58.8%) were men with a mean age of 68 years (± 12.6). Heart failure was the final diagnosis in 53% of dyspneic patients (HF group, *n* = 371).

**Table 1:** Characteristics of study population

	Total *n* = 700	HF group *n* = 371	Non-HF group *n* = 329	*p*
Age (years), mean(SD)	68 (20)	70(24)	65(20)	< 0.001
Sex ratio (male/female)	1.43	1.13	1.90	< 0.001
Past medical History, *n* (%)				
COPD	151 (21.6)	44 (11.8)	107 (32.5)	< 0.001
Asthma	21 (3)	10 (2.7)	11 (3.3)	0.47
Hypertension	346 (49.4)	225 (60.6)	121(36.7)	< 0.001
Diabetes mellitus	287 (41)	188 (50.6)	99 (30)	< 0.001
Chronic heart Failure	175 (25)	128 (34.5)	47 (14.2)	< 0.001
Coronary artery disease	133 (19)	98 (26.4)	35 (10.6)	< 0.001
Treatments				
Angiotensin converting enzyme (ACE) inhibitors	193 (27.5)	176 (47.6)	17 (5.2)	< 0.001
Diuretics	167 (23.8)	158 (42.7)	9 (2.6)	< 0.001
Beta blockers	50 (7.1)	42 (11.3)	8 (2.4)	< 0.001
β 2 mimetics	102 (14.5)	64 (17.2)	38 (11.7)	0.03
Steroids (inhaled)	53 (7.5)	23 (6.3)	30 (9)	0.14
Aspirin	124 (17.7)	89 (23.9)	35 (10.7)	< 0.001
Chest X-ray, *n* (%)				
Cardiomegaly^a^	368 (52.5)	253 (68.2)	115 (35)	< 0.001
Interstitial edema	484 (69)	279 (75.2)	205 (62)	0.004
Vascular pulmonary redistribution	290 (41.4)	208 (56)	82 (25)	< 0.001
Pleural effusion	227 (32.5)	144 (39)	81 (25)	0.003
Atrial fibrillation	154 (22)	98 (26.4)	56 (17)	0.003
LV ejection fraction, mean (SD)	51 (14)	44 (13)	59 (10)	< 0.001
BNP, pg/ml, median [IQR]	216 [68–548]	458[215–771]	62[25–162]	< 0.001

*HF* heart failure, *IQR* interquartile range, *COPD* chronic obstructive pulmonary disease, *LV* left ventricle, *BNP* brain natriuretic peptide

^a^Cardiomegaly: cardiothoracic ratio > 0.5

**Fig. 4:**
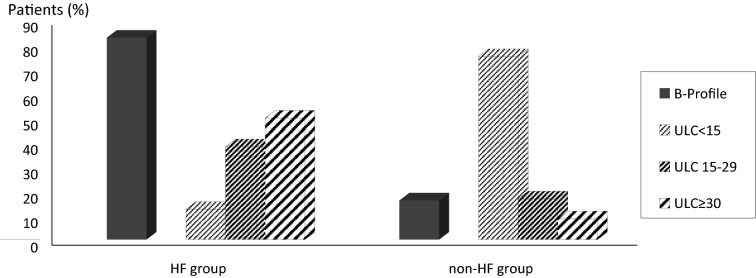
Distribution of B-profile pattern and ultrasound lung comets (ULC) score between the heart failure (HF) and non-HF groups. *p < 0.001* between HF and non-HF groups for B-profile pattern*; **p value < 0.001 *between HF and non-HF groups for ULC score*

**Fig. 5:**
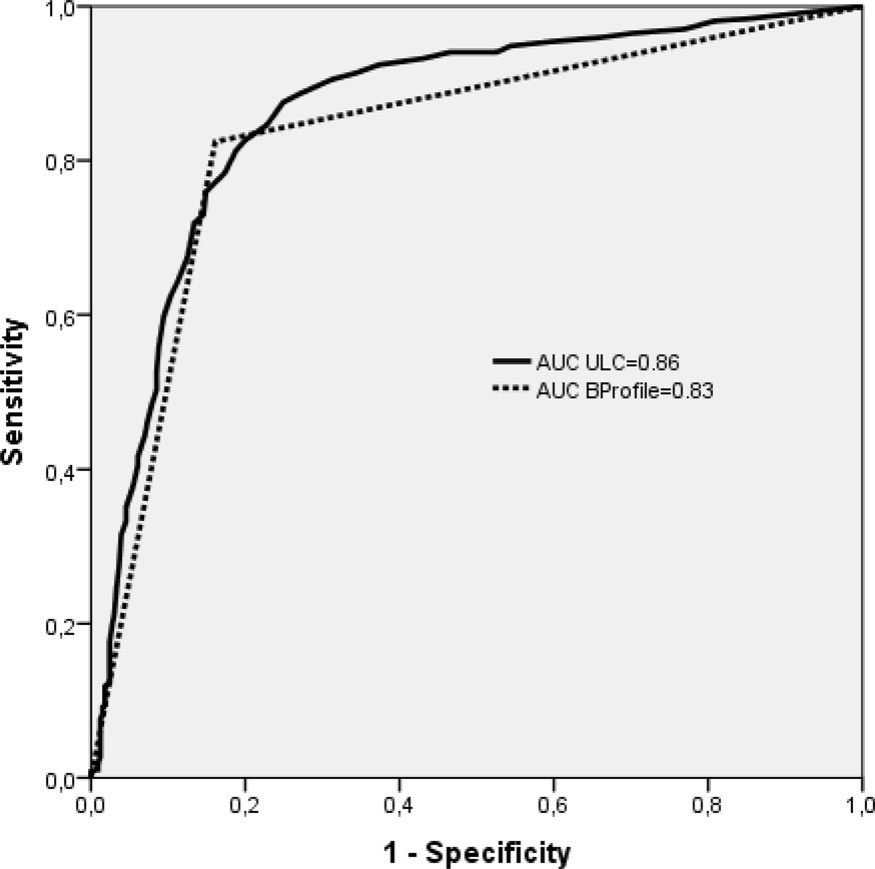
Receiver operating characteristic (ROC) curve for ultrasound lung comets (ULC) score and B-profile pattern

**Fig. 6:**
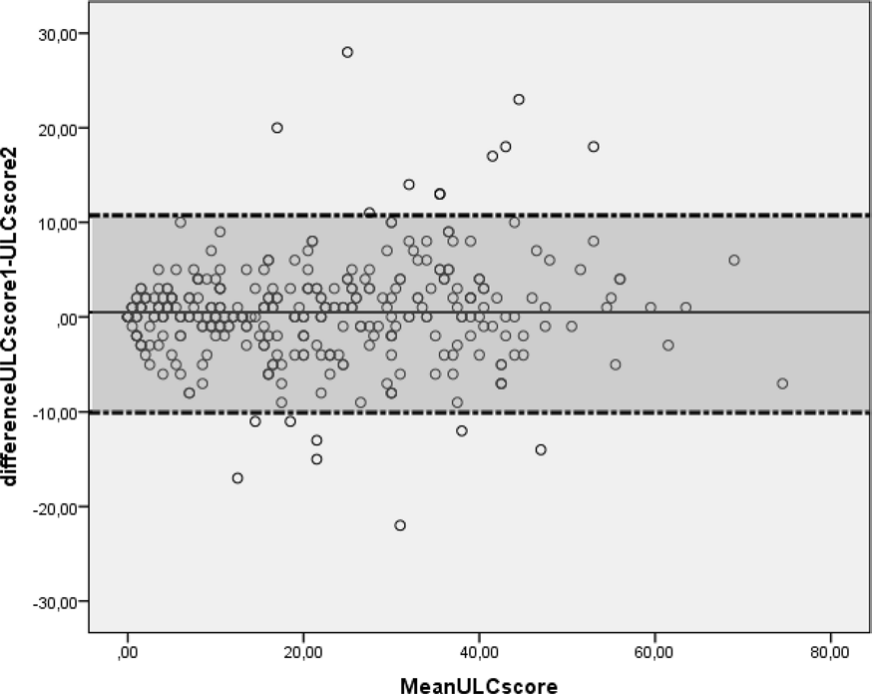
Bland and Altman plot for ultrasound lung comets score. ULCscore1 denotes ultrasound lung comets score measured by the first operator; ULCscore2 denotes ultrasound Lung comets score measured by the second operator of the same pair of sonographers; shaded area denotes agreement limits

The most common etiology of dyspnea in non-HF group (*n* = 329) was chronic obstructive pulmonary disease exacerbation (*n* = 149), pneumonia (*n* = 57), pulmonary embolism (*n* = 19), and acute asthma (*n* = 12). The mean B-lines score was 29 ± 9 in HF group and 8 ± 3 in non-HF group. The difference was statistically significant (*p* < 0.001). In HF group, the B-lines score was suggestive of CHF (B-lines score ≥ 15) in 325 patients (87.6%). In the same group, B-profile pattern was present in 306 patients (82.5%). The difference in patients’ distribution between HF and non-HF groups according to B-profile and B-lines classes is summarized in Fig. 4. This difference was statistically significant (*p* < 0.001). The discriminating power of B-lines score and B-profile pattern was good as assessed by area under ROC curve of 0.86 (95% CI 0.83–0.89) and 0.83 (95% CI 0.80–0.86), respectively, for B-lines score and B-profile pattern (*p* = 0.91) (Fig. 5). Performance of B-lines score at a cut-off = 15 showed that sensitivity, specificity, negative predictive value and positive predictive value of the two models were similar with trends to a moderately higher sensitivity for B-lines score compared to B-profile pattern (87.6% versus 82.5%) and lower specificity (74.7% versus 83.9%) (Table 2). Agreement between residents in the determination of CHF diagnosis was excellent for both models as demonstrated by kappa agreement index value of 0.81 and 0.85, respectively, for B-lines score and B-profile pattern. For B-lines scoring, there is a good agreement between residents’ interpretation as shown in the Bland and Altman plot (mean differences between B-lines scores = 0.49 ± 0.22, p: not significant) (Fig. 6).

**Table 2:** Ultrasound lung comets (ULC) score and B-profile pattern accuracy

	ULC score^a^	B-profile pattern
	%[95% confidence interval]	
Sensitivity	87.6 [83.8–90.6]	82.5 [78.3–86]
Specificity	75.1 [70.1–79.4]	83.9 [79.5–87.5]
Positive predictive value	79.9 [76–83.7]	85.2 [81.6–88.9]
Negative predictive value	84.3 [80.1–88.5]	80.9 [76.8–85.1]
Positive likelihood ratio	3.51 [2.90–4.25]	5.12 [3.98–6.58]
Negative likelihood ratio	0.165[0.12–0.21]	0.20 [0.16–0.26]

^a^Cut-off = 15

## Discussion

Our study has shown study that EM residents can be significantly aided to establish the diagnosis of CHF after a short and accelerated ultrasonographic B-lines assessment training, with an excellent inter-rater agreement in patients admitted for acute dyspnea.

Among the many potential underlying causes of acute dyspnea, CHF is one of most common and challenging etiologies [16]. Among patients presenting to the ED with CHF, over 80% are admitted to the hospital, making it the most common reason for admission and a significant financial burden on the health care system. Despite this high prevalence, the standard workup for acute shortness of breath in the ED is non-specific and often fails to differentiate CHF from conditions such as chronic obstructive pulmonary disease exacerbation [17]. This distinction is essential as inappropriate management has been shown to affect negatively the morbidity and mortality. Overall, approximately 20% of patients presenting to the ED with dyspnea are misdiagnosed and treated inappropriately [18]. In fact, substantial diagnostic uncertainty is inevitable when relying only on traditional clinical findings [19]. Lung ultrasonography, once considered inconceivable, is increasingly considered as a bedside imaging tool for evaluating pulmonary congestion [20]. A recent systematic review showed that B-lines study is highly accurate in the diagnosis of acute heart failure with an area under ROC of 0.91, a sensitivity of 0.90 and a specificity of 0.93 [21]. Of note, many of the studies included in this review had small sample sizes and were performed in settings other than EDs, even though our results are consistent with the findings of this meta-analysis. Importantly, our study is the largest in demonstrating the accuracy of B-lines study performed by residents with no previous experience of ultrasound techniques. Similar results were reported by Bedetti et al. in a smaller sample size study [22]. In addition, in their estimations of the sensitivity and specificity, Chiem et al. showed results close to ours, but slightly lower than those reported in previous studies [5, 12, 23]. Of note, all these studies included non-expert operators. It is possible that, with sustained and more supervised practice, these novice trainees would improve significantly their performance**.**

The second important objective of the present study was to assess the reproducibility of B-lines. Available evidence regarding inter-observer agreement reveals that B-lines study has a good is reproducibility [7, 23, 24]. It should be highlighted that most studies assessing reproducibility were based on retrospective LUS imaging review performed longtime after the first LUS testing. B-lines is a dynamic phenomenon that can be influenced with number of technical and pathologic factors [25]. Consequently, reproducibility should be assessed without delay between pairs of LUS examinations and ideally in the same conditions. In the present study, we minimized this time between each pair of operators testing (one immediately followed the other). Moreover, we demonstrated the excellent inter-observer agreement of B-lines study by using two different models, the B-lines scoring system and the B-profile pattern which reinforces the validity of the results.

### Limitations

Our study has some limitations. First, the study was conducted in academic EDs and the same evaluation in another setting may show different results. Second, since only hospitalized patients were considered eligible for the study purpose, a selection bias could not be excluded and our results may not be applicable to patients with milder symptoms. Third, some of our patients received specific heart failure treatment (intravenous diuretics, nitrates, CPAP) before undergoing LUS test, which could improve lung congestion, B-lines number would be reduced and this would probably underestimate the sensitivity of B-lines testing. Lastly, it is not clear whether introduction of LUS in routine clinical practice would influence medical decision-making and change patients’ prognosis? It is not possible for us to give a clear answer to this question; it is above the scope of the present study. Nonetheless, the fact that LUS can help to identify rapidly the diagnosis of CHF, this would give to physicians more confidence in choosing the most appropriate and effective treatment. Fourth, the training course of residents is limited in our study to 2 h; this could be insufficient to be comfortable to practice LUS. However, according to a recent meta-analysis in clinical lung ultrasound, the learning time spent in the different included studies ranged from 30 min sessions to 2.5 h sessions [26]. Similar brief durations reported by Noble et al. (1 h) resulted in a significant improvement of image recognition skills for physicians without previous ultrasound experience. Moreover, a recent study by Gargani et al. showed that even web-based training in lung ultrasound can be a highly effective approach for training inexperienced operators [27].

In summary, the present study demonstrated that point-of-care B-lines study in the hand of non-expert residents is a reliable and reproducible technique. It can improve the identification of CHF in ED patients with undifferentiated dyspnea. Our results, if confirmed by other larger prospective high-quality studies, have potentially significant clinical implications. Being a rapid technique with high accuracy in the diagnosis of cardiogenic dyspnea, B-lines study could be suitable in departments with lack of technical and human resources.

## Data Availability

The datasets used and/or analyzed during the current study are available from the corresponding author on reasonable request.

## Abbreviations

ED: Emergency Department
CHF: Congestive heart failure
M/F: Male/female
LUS: Lung ultrasound
SD: Standard deviation
IQR: OR interquartile range
HF: Heart failure
LR: Likelihood ratios
AUC: Area under curve
